# Improving the learning of clinical reasoning through computer-based cognitive representation

**DOI:** 10.3402/meo.v19.25940

**Published:** 2014-12-16

**Authors:** Bian Wu, Minhong Wang, Janice M. Johnson, Tina A. Grotzer

**Affiliations:** 1 Department of Educational Information Technology, East China Normal University, Shanghai, China; 2 KM&EL Lab, The University of Hong Kong, Hong Kong, China; 3 Faculty of Education, The University of Hong Kong, Hong Kong, China; 4 Faculty of Medicine, The University of Hong Kong, Hong Kong, China; 5 Harvard Graduate School of Education, Harvard University, MA, USA

**Keywords:** clinical reasoning, problem solving, knowledge construction, cognitive representation, computers/technology

## Abstract

**Objective:**

Clinical reasoning is usually taught using a problem-solving approach, which is widely adopted in medical education. However, learning through problem solving is difficult as a result of the contextualization and dynamic aspects of actual problems. Moreover, knowledge acquired from problem-solving practice tends to be inert and fragmented. This study proposed a computer-based cognitive representation approach that externalizes and facilitates the complex processes in learning clinical reasoning. The approach is operationalized in a computer-based cognitive representation tool that involves argument mapping to externalize the problem-solving process and concept mapping to reveal the knowledge constructed from the problems.

**Methods:**

Twenty-nine Year 3 or higher students from a medical school in east China participated in the study. Participants used the proposed approach implemented in an e-learning system to complete four learning cases in 4 weeks on an individual basis. For each case, students interacted with the problem to capture critical data, generate and justify hypotheses, make a diagnosis, recall relevant knowledge, and update their conceptual understanding of the problem domain. Meanwhile, students used the computer-based cognitive representation tool to articulate and represent the key elements and their interactions in the learning process.

**Results:**

A significant improvement was found in students’ learning products from the beginning to the end of the study, consistent with students’ report of close-to-moderate progress in developing problem-solving and knowledge-construction abilities. No significant differences were found between the pretest and posttest scores with the 4-week period. The cognitive representation approach was found to provide more formative assessment.

**Conclusions:**

The computer-based cognitive representation approach improved the learning of clinical reasoning in both problem solving and knowledge construction.

Clinical reasoning, the sum of clinical problem solving and diagnostic reasoning, is the foundation of professional clinical practice. Clinical reasoning is difficult to teach and learn as it involves complex processes in collecting evidence about the problem, analyzing and evaluating the data, and formulating appropriate hypotheses (1). Competence in clinical reasoning requires extensive exposure to case examples with deliberate practice and adequate supervision (2). In addition to internship programs that consist of close expert supervision while interacting with patients, case-based sessions in the classroom are widely used in medical schools. They are relevant to the problem-based learning model, which allows learners to work in groups, solve clinical problems, and reflect on the experience, while the teacher facilitates the learning process rather than providing knowledge (3). Given that clinical problem solving through internship programs or classroom sessions faces time and resource constraints (4), computer simulations and virtual reality techniques have been increasingly used as alternative approaches to situating learning in problem contexts (5), though there is concern about a limited number of cases provided in these applications due to the programming costs (6).

Problem-based learning is found to be effective in motivating learners, improving their reasoning and communication skills, and fostering their abilities to cope with uncertainty and self-directed learning (7–11). Meanwhile, because clinical problem solving is characteristic of higher-order cognitive activities involving exploration with incomplete information and interactive components, scaffolding or supporting problem-based learning of novices therefore becomes important (12, 13). Given a limited resource of experts for supervision of novices, intelligent tutoring systems that provide computer-generated, personalized feedback to learners have been increasingly explored for education in many domains including learning clinical reasoning (14). The feedback from the computer is mainly generated by monitoring the learner's performance and evaluating the performance based on expert knowledge specified in the system. The development of such applications is under further improvement, and their impact on education is under investigation.

Recent studies on supporting clinical problem solving have extended the focus from intelligent tutoring to making complex cognitive processes involved in clinical problem solving accessible to learners and instructors. For example, thinking-aloud protocols that externalize clinical reasoning and thinking processes in an explicit format have been used for understanding the performance of experts (15) and for teaching clinical reasoning. Clinical educators who are experienced clinicians find it difficult to explain and teach clinical reasoning because these processes have become reflexive in their way of thinking. Making complex reasoning processes visible is found to be effective in fostering reflective teaching of clinical reasoning (16). Further investigations are expected to examine the effects of such approaches on student learning of clinical reasoning.

In addition to clinical reasoning and problem-solving processes, revealing the network of understanding underlying the problems and the update of the knowledge network based on accumulated experience have received increased attention (17). Problem-solving practice is found to train more routine experts who follow rigid protocols than adaptive experts who continually learn and update their knowledge based on experiences with new problems (18, 19). In problem-based learning contexts, many learners have difficulties separating general knowledge from the problem context and transferring it to new problems (20, 13). Studies on expertise development highlight the importance of both systematic practice and progressive crystallization of knowledge (21).

The attention to revealing the network of knowledge underlying the problems has been reflected in the use of concept maps to represent the theoretical knowledge that underpins clinical practice (22, 23). Existing studies on concept maps have focused on analysis of self-constructed concept maps for reflection and assessment (24). Students using concept maps were found to perform better in problem solving (25) and basic science (26) examinations. More studies are needed to examine how the construction of a concept map can be guided in a way that fosters systemic thinking and meaningful understanding. Another type of cognitive tool related to problem solving is argument map, a visual representation of an argument's structure in informal logic involving fact, claims, explanations, evidence, and rebuttals. Computer-assisted argument mapping is assumed to be a promising approach to facilitating complex problem solving, although there is little evidence in the literature (27, 28).

This study aims to investigate how computer-based cognitive representations can be used in an effective way that fosters systemic thinking and reasoning processes in learning with complex problems. The study presents a computer-based cognitive representation approach that externalizes and facilitates the complex processes in the learning of clinical reasoning. The approach is operationalized in a computer-based cognitive representation tool implemented in an e-learning system. e-Learning provides clear advantages to education in terms of flexibility and cost effectiveness in delivery of learning programs, and may address the concerns of time and resource constraints in traditional problem-based learning in the classroom (29). Nephrology, the study of kidney function and problems, was selected as the learning subject because many students found clinical reasoning or practice in this area to be challenging.

The purpose of this study is to examine: 1) how a computer-based cognitive representation approach can be designed and implemented for learning clinical reasoning, and 2) the effectiveness of the approach in supporting the learning of clinical reasoning. The clinical reasoning performance in this study concerns learners’ performance not only on solving clinical problems by capturing critical data, formulating hypotheses, and reasoning with justifications, but also on constructing knowledge from the problems by revealing a network of key concepts in the problem domain. More details are presented in the measures of the study.

The following three research questions are explored in the present study.

1. Will the proposed computer-based cognitive representation approach improve learners’ overall learning outcomes reflected in traditional examinations?
2. Will the proposed computer-based cognitive representation approach improve learners’ clinical reasoning performance reflected in their learning products or cognitive maps?
3. Will learners perceive that their problem-solving and knowledge-construction abilities are improved by using the proposed computer-based cognitive representation approach?

## Methods

### Design

The computer-based cognitive representation approach studied here involves argument mapping to externalize the problem-solving process and concept mapping to reveal the knowledge constructed from the problems. Learners articulate the problem-solving process into critical information (data nodes), generate hypotheses (hypothesis nodes), and reason with justifications (reasoning links) in an argument map. Meanwhile, learners represent the conceptual knowledge underlying the problem-solving process into a set of concepts and their relationships (including causal, hierarchical, and cross-link) in a concept map. Furthermore, learners are encouraged to connect the nodes in the concept map with relevant nodes in the argument map to indicate the connection between problem solving and knowledge construction, that is, to reveal the knowledge applied to or acquired from the problem-solving practice.

**Fig. 1:**
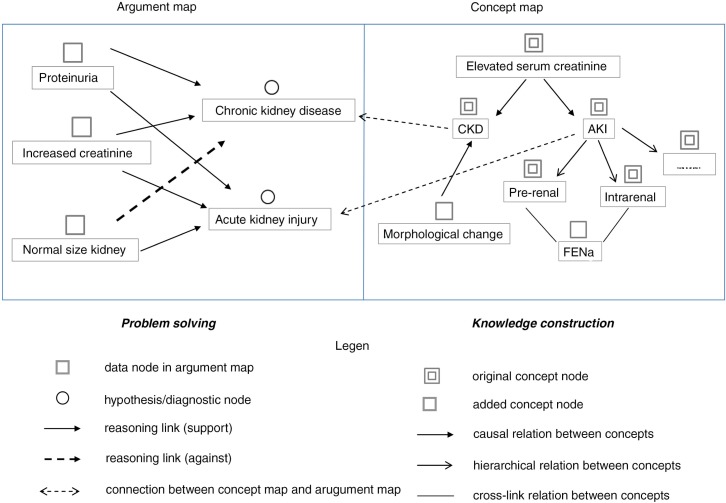
Cognitive tool.

An example of using this approach for learning clinical reasoning is shown in Fig. 1. The patient was observed to have proteinuria and increased serum creatinine. Based on the two symptoms, the learner recalled relevant knowledge. Elevated serum creatinine might be caused by chronic kidney disease (CKD) or acute kidney injury (AKI) in general, as represented in the concept map. Accordingly, two hypotheses CKD and AKI were generated. The CKD hypothesis was rejected, and the AKI hypothesis was supported with the further information of a normal sized kidney. Such hypothesis generation and justification processes were informed by the knowledge about CKD and AKI, and were explicitly represented by the links between the argument map and the concept map. During the problem-solving and reasoning process, the learner recalled other knowledge relevant to CKD and AKI. As represented in the concept map, CKD may cause morphological change in kidney; AKI may cause prerenal and intrarenal diseases, and fractional excretion of sodium can be used for differentiation.

The proposed computer-based cognitive representation approach was implemented in an e-learning system, which consisted of three main functions: 1) a simulated exploratory problem context for learners to interact with the problem and obtain relevant information, 2) a cognitive representation tool that helps learners to articulate and represent their problem-solving and knowledge-construction processes, and 3) scaffolding and coaching support to facilitate the learning process.

**Fig. 2:**
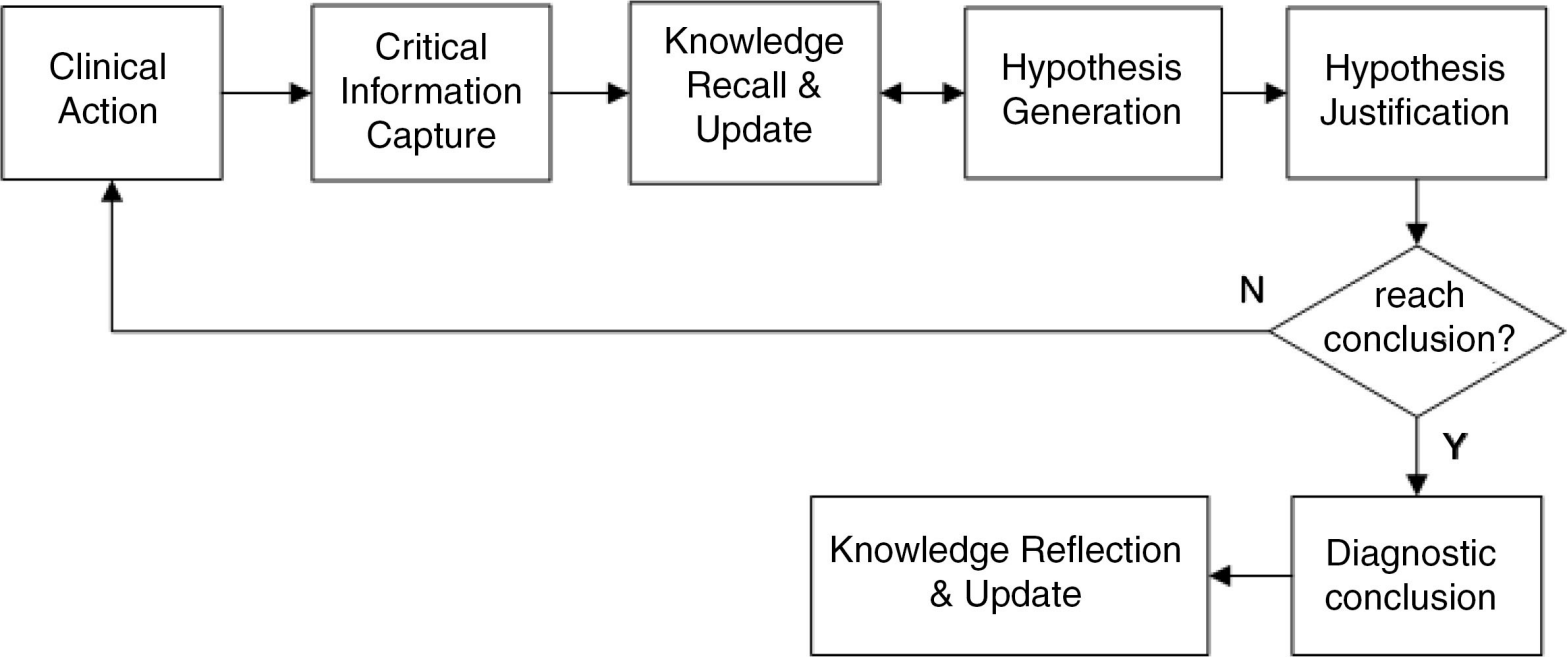
Learning flowchart.

The *simulated problem context*, working as an interactive virtual patient, allows learners to select a problem case, receive its initial information, and activate hypothesis-led clinical actions with the case to obtain further information. The case information was categorized into patient history, physical examinations, lab tests, imaging records, patient state, and prescription history. The *cognitive representation tool* enables learners to articulate and represent their problem-solving and knowledge-construction processes for each case into a dual map, as shown in Fig. 1. To facilitate the learning process, computer-based *scaffolding and coaching support* were provided. As shown in Fig. 2, a learning flowchart that decomposes the complex learning process into a set of iterative tasks was provided to scaffold the general learning process. The iterative tasks included: perform clinical actions, identify critical information, recall and update knowledge, generate hypotheses, justify hypotheses, and make a diagnostic conclusion. In addition to scaffolding the general learning process, case-specific, personalized guidance was provided to support individual learning.

The proposed approach was informed by the cognitive apprenticeship theory and its cognitive strategies including exploration, articulation, reflection, modeling, coaching and scaffolding (30), which emphasize that complex tasks should be situated in authentic contexts and the thinking processes involved must be made visible for learners to observe, enact, and practice.

### Participants

A face-to-face introduction to the learning system and the learning approach was provided to 50 senior students taking a residential course in a medical school in east China. The students were in different years (Year 3 to Year 5) of their 7-year medical school curriculum. They had completed the courses for fundamental medical knowledge in their earlier years study, but had little practice with clinical problems. Twenty-nine of them gave informed consent to participate in the study after the introduction session. Their participation in this project was fully voluntary, that is, based on their interest and time availability instead of course requirements. The study received ethical approval from the Human Research Ethics Committee for Non-Clinical Faculties of the researchers’ university. According to their responses to a demographic questionnaire, 65.5% of the participants were females and 34.5% were males. Most of them (65.5%) were in Year 4, and the rest were in Year 3 and Year 5. Most of them had intermediate (41.4%) to good (41.4%) computer skills.

### Procedure

An online learning program for clinical reasoning was delivered using the developed system. The learning program included five kidney disease cases of similar difficulty, a sample case for demonstration and practice, and four cases for independent study by the learners. Two domain experts and one instructor from the school assisted in selecting and adapting the cases from clinical practices and academic references. The participants were provided with a face-to-face session and online videos demonstrating the use of the proposed approach. The sample case was provided for demonstration and pre-study practice by students to gain familiarity with the learning approach. Self-study with the four cases started 1 week later when all the participants were able to use the learning program.

Learners were asked to complete the learning tasks in their free time within a 4-week period. They were asked to pace themselves, and spend 5 hours per week on the program. They proceeded through each case, following the learning flowchart, and they represented key elements of the learning process in a dual map for each case. During the learning period, the instructor was available to provide limited support when needed, and students were free to utilize learning materials or resources external to the system. Online forums were provided for open discussion.

For each case, learners were instructed to go through the following steps:

*Interact with the problem*. The learner accesses a clinical case to elicit initial information (such as chief complaint of the patient) and perform clinical actions with the case to obtain further information.

*Identify critical information*. The learner identifies the critical information of the case, and generates one or more corresponding data node in the argument map.

*Recall and update knowledge*. The case information triggers the learner's recall of relevant knowledge to solve the problem. The knowledge can be represented as a set of linked concepts in the concept map, which can be further updated throughout the learning process.

*Generate hypotheses*. Based on the case information and relevant knowledge, the learner generates one or more hypotheses, and represents them as hypothesis nodes in the argument map.

*Justify hypotheses*. The learner goes through each hypothesis and justifies or rejects it by creating reasoning links between the hypothesis nodes and relevant data nodes.

*Diagnostic conclusion*. After justifying all the hypotheses, the learner draws a diagnostic conclusion, and represents it as a diagnostic node in the argument map. In most cases, iterative clinical actions are needed to explore additional information before a diagnostic conclusion can be reached.

### Measures

According to the literature, assessment of learning in problem-solving contexts should consider problem-solving skills as well as knowledge learning issues (31–34). The former focuses on the reasoning abilities involving not only the number and accuracy of diagnosis, but also the capture of critical information and logical steps in reasoning. The latter refers not just to the knowledge of separate concepts, but also to the integration of relevant ideas and concepts in the problem domain, which can be reflected in a knowledge profile involving core concepts and their relationships (33, 35). To assess a knowledge profile represented in a concept map, individual concepts and their relationships or links are commonly analyzed (36–38).

In this study, the participants completed two knowledge tests (pretest and posttest) with questions comparable to those used by the medical school. Different questions but of similar difficulty were used for pretest and posttest. Each test included three multiple choice, ten extended matching, and four essay questions. The scores ranged from 0 (incorrect) to 4 (full credit) for each question, with a test range of 0–68 rescaled to the range 0 and 1. The essay questions were assessed on a five-level scale including 0: little argument and evidence; 1: argument with irrelevant evidence; 2: argument supported by limited evidence; 3: argument supported by more evidence; 4: argument supported by sufficient evidence. The test papers were scored by one instructor, who was blind to student identification and test information (i.e., whether the test was pretest or posttest).

The learning products, that is, dual maps generated by learners for the first and the last cases were assessed, blind to testing sequence, by two domain experts based on a set of predefined rubrics adapted from the aforementioned prior studies. The assessment focused on five aspects of performance: how students observed critical information, formulated hypotheses, performed reasoning for justification (31, 32, 34), and generated concepts and concept relationships (36–38). Accordingly, the rubrics involved quantity and quality of the five aspects, that is, data nodes, hypothesis nodes, and reasoning links in the argument map, and concept nodes and concept relations in the concept map, as shown in Table 1. Performance in each aspect was scored on a five-level scale between 0 (lowest) and 1 (highest). The overall dual-mapping score was the average score for the five aspects. The links between the argument map and the concept map built by the learner were also analyzed.

**Table 1:** Rubrics for assessing the learning product

Dimensions	Elements	Descriptions
*Problem-solving process (argument map)*		
Identified critical information	Data nodes in the argument map	Identify critical data from the patient information 0: no critical, well described data nodes 1: mostly critical, well described data nodes
Formulated hypotheses	Hypothesis nodes in the argument map	Formulate hypotheses 0: no plausible hypotheses 1: plenty of plausible, differential diagnostic hypotheses in a strategic sequence of from general to more specific
Performed reasoning	Reasoning links in the argument map	Perform reasoning links to support/refute hypotheses 0: no justified, incorrect reasoning links 1: sufficient well-justified reasoning links
*Knowledge-construction process (concept map)*		
Generated concepts	Concept nodes in the concept map	Trigger concept nodes from identified critical information 0: none or irrelevant concept nodes 1: plenty of closely-related, problem-solving-oriented concept nodes
Generated relations between concepts	Concept relations in the concept map	Construct concept relations among concept nodes in the concept map 0: none or incorrect concept relations 1: plenty of well-organized, thought-provoking, and cross-linked concept relations

At the end of the learning program, a survey was administered with learners to collect their perceived learning gains with regard to problem-solving and knowledge-construction abilities. The measuring items were adopted from the Student Assessment of their Learning Gains instrument (39), which has been internationally validated and widely used (40). Internal consistency analysis using Cronbach's alpha confirmed that all subscales were reliable (0.85 for problem-solving ability, 0.79 for knowledge-construction ability). Moreover, semi-structured written interviews were arranged to collect students’ responses to two open-ended questions: 1) advantages and disadvantages of the learning system, and 2) suggestions for improvement of the learning system. The collected responses were summarized.

## Results

### Test result

The paired-sample *t*-test indicated no significant difference between the pretest and posttest scores, albeit a slight increase in the mean score (pretest mean=0.24; posttest mean=0.29, *p*=0.136).

### Learning products

All the participants completed the learning tasks for the four cases. Dual maps generated by learners from the first and the last cases were blind coded by the two domain experts. The inter-rater reliability of assessment by the two raters was 0.91.

The descriptive statistics and paired-sample *t-*tests are presented in Table 2. A significant improvement in the overall performance was found (first case mean=0.24; last case mean=0.38; first to last case: *t*(28)=5.72, *p*=0.000), with the effect size^1^ of 1.17, indicating substantial progress from the first to the last case. As the dual-mapping performance in the five aspects were concerned, there was a significant improvement in all aspects except reasoning links. The learners’ problem-solving performance (reflected in data nodes, hypothesis nodes, and reasoning links in the argument map) was better than their knowledge-construction performance (reflected in concept nodes and concept relations in the concept map) for both the first and the last cases. Furthermore, the knowledge-construction performance presented a larger variation among the participants in the last case than in the first case.

**Table 2:** Dual-mapping scores for the first and last cases (scores range from 0 to 1)

	First case		Last case		Paired-sample *t-*tests		
	
	Mean	Standard deviation	Mean	Standard deviation	*t*	*df*	*p*
DaN	0.44	0.16	0.58	0.12	3.92	28	0.002
HyN	0.25	0.15	0.35	0.13	2.80	28	0.017
ReL	0.23	0.17	0.35	0.20	1.73	28	0.111
CoN	0.17	0.16	0.35	0.31	3.45	28	0.005
CoR	0.13	0.17	0.23	0.25	2.80	28	0.017
Overall	0.24	0.11	0.38	0.14	5.72	28	<0.001

DaN: data nodes; HyN: hypothesis nodes; ReL: reasoning links; CoN: concept nodes; CoR: concept relations.

There was a significant improvement from the first to the last case (*t*(28)=2.67, *p*=0.045) in the number of connections from problem solving to knowledge construction (represented by links from argument map to concept map), as shown in Table 3. The improvement indicated that learners were able to consolidate the connection from problems to knowledge in more occasions after the study. But no significant difference was found in the number of connections from knowledge construction to problem solving (represented by links from concept map to argument map).

**Table 3:** Numbers of connections from problem solving to knowledge construction (PS2KC) and from knowledge construction to problem solving (KC2PS) in the first and last cases

	First case		Last case		Paired-sample *t*-tests		
	
	Mean	Standard deviation	Mean	Standard deviation	*t*	*df*	*p*
PS2KC	2.33	1.50	3.83	1.79	2.67	28	0.045
KC2PS	2.17	1.17	2.50	1.39	0.79	28	0.465

### Survey and interviews results

Learners reported their perceived learning gains in both problem-solving and knowledge-construction abilities to be close to moderate, as shown in Table 4.

**Table 4:** Perceived learning gains (5-point Likert scale: 0 represented ‘no progress’ and 4 represented ‘substantial progress’)

	Problem-solving ability	Knowledge-construction ability
Mean	1.87	1.97
Standard deviation	0.88	0.98

The interview results showed that most learners regarded the proposed learning approach to be useful and innovative, but suggested that the interfaces of the system could be improved and more learning guidance could be provided at the beginning of the learning program. The instructor and domain experts offered their spontaneous comments that the proposed approach offered students meaningful and useful learning experience and empowered them to become self-directed and engaged. They suggested the incorporation of the approach into medical education programs after refining some interfaces.

## Discussion

This study considered a computer-based cognitive representation approach to learning clinical reasoning and examined its effects on improving problem-solving and knowledge-construction performance. First, the approach was found to be promising in improving the learning of clinical reasoning as reflected by the findings relevant to the three research questions (whether it would improve learners’ outcomes on traditional examinations, learning products, and cognitive maps, as well as their perceptions of their problem-solving and knowledge-construction abilities). Although no significant differences were found between the pretest and posttest scores with the 4-week period, a significant improvement was found in students’ learning products from the beginning to the end of the study, consistent with students’ report of close-to-moderate progress in problem-solving and knowledge-construction abilities. The result is consistent with findings from previous studies in that the outcomes are mixed and the learning gains cannot be fully reflected in traditional examination scores (32, 7).

Second, different from the summative assessment used in traditional examinations, the cognitive representation approach studied here offers insight into the formative assessment of learning through problem-solving practice. The results show that learners made a significant improvement from the first case to the last case in both problem-solving and knowledge-construction performances; learners performed better in problem solving than in knowledge construction for both the first and the last cases; learners made more connections from problem solving to knowledge construction in the last case than in the first case; and the knowledge-construction performance varied more than the problem-solving performance among learners in the last case. It seems that knowledge construction, compared with problem solving, is more challenging to most learners and difficult to improve in a short period of time.

There are some limitations of the study. First, preliminary findings from a small number of participants may not be sufficient to claim the effectiveness of the approach for a broader population. Second, the study was conducted in a local context. There may be cultural influences which limit the generalizability of the findings. Third, conclusions drawn from this study are limited by the lack of a control group. Considering the complexity of real educational settings and the nature of learning, it is not easy to precisely attribute any learning outcome to a single teaching and learning medium. A control group design will be carefully implemented in future studies.

## Conclusion

Clinical problem solving or diagnostic reasoning is the core of medical practice, where deliberate practice and progressive crystallization of knowledge are the focus. Although learning through problem-solving practice has been widely adopted in medical education, problem-solving and reasoning processes tend to be tacit and difficult to master. Moreover, construction of systemic knowledge from problems is found to be more important in development of clinical expertise, but difficult to realize. This study attempted to address this challenge by proposing a computer-based cognitive representation approach that externalizes and facilitates the complex cognition in the learning of clinical reasoning. The results demonstrated the effects and potential of the approach in improving problem-solving and knowledge-construction performance in the learning of clinical reasoning contexts. The findings also provided insight into computer-assisted instructional design and assessment for learning through clinical problem solving. Further work will address the limitations of the present study.

## Footnotes

1 $d=\frac{x_{1}-x_{2}}{s}$, where $s=\sqrt{\frac{\left(n_{1}-1\right)s_{1}^{2}+\left(n_{2}-1\right)s_{2}^{2}}{n_{1}+n_{2}}}$ d: Cohen's d, i.e., effect size. x_1_, x_2_: mean for the first case and the second case. s_1_, s_2_: standard deviation for the first case and the second case. n_1_, n_2_: sample size for the first case and the second case.
